# Psychosis in multiple sclerosis—a narrative review of an underestimated, but clinically significant problem

**DOI:** 10.1007/s00702-026-03180-6

**Published:** 2026-05-14

**Authors:** Piotr Olejnik, Antonio Prisco, Marco Menchetti, Kaja Kasarełło

**Affiliations:** 1 Department of Experimental and Clinical Physiology, Laboratory of Centre for Preclinical Research, Medical University of Warsaw, Warsaw, 02-097 Poland; 2 Department of Neurology, Medical University of Warsaw, Warsaw, 02-097 Poland; 3 Department of Medical and Surgical Sciences, University of Bologna, Bologna, 40126 Italy; 4 Department of Biomedical and Neuromotor Sciences, University of Bologna, Bologna, 40126 Italy

**Keywords:** Multiple sclerosis, Psychosis, Psychotic disorders, Neurotransmission disruption, Psychosis treatment

## Abstract

Multiple sclerosis (MS) is a chronic autoimmune inflammatory disease of the central nervous system characterized by immune-mediated demyelination and subsequent axonal injury and neurodegeneration. Traditionally, the pathogenesis of MS has been attributed primarily to autoreactive immune responses targeting myelin sheaths antigens. However, growing evidence highlights the role of neurotransmitter dysregulation, particularly involving glutamate, acetylcholine, and dopamine pathways, in the development and progression of MS. These alterations in neurotransmitter systems may be especially relevant to the emergence of neuropsychiatric manifestations, including psychosis, which appears to occur more frequently in individuals with MS than in the general population. Also, some of the medications used in MS treatment might cause psychosis itself. Despite increasing recognition of MS-associated psychosis (P-MS), its underlying mechanisms remain poorly understood. This narrative review aims to provide a comprehensive overview of this often underestimated and potentially underdiagnosed clinical phenomenon. The current literature was reviewed with regard to the prevalence and risk factors of P-MS, as well as proposed pathophysiological mechanisms, including inflammatory, neurochemical, and structural contributors. Clinical presentations and differential diagnostic considerations are also discussed. Furthermore, evidence-based strategies for the management of P-MS are examined, with emphasis on the importance of interdisciplinary collaboration between neurologists and psychiatrists. By synthesizing current knowledge, this review seeks to raise awareness of P-MS as a clinically significant comorbidity that warrants further research and improved diagnostic and therapeutic approaches.

## Introduction

Multiple sclerosis (MS) is a chronic autoimmune disorder affecting central nervous system (CNS), associated with inflammation of the myelin sheaths, leading to demyelination and subsequent axonal damage and degeneration of neurons (Nicol et al. 2015; Thompson et al. 2018). The etiology of MS is complex, involving genetic and environmental aspects, and to date remains to be fully understood (Thompson et al. 2018). Generally, it is accepted that MS develops in genetically susceptible individuals exposed to certain environmental factors. For instance, those carrying the HLA-DRB1*15:01 allele are at a threefold higher risk of developing MS compared to individuals who do not carry this gene (Thompson et al. 2018; Oh et al. 2018). Amongst the environmental factors, Epstein–Barr virus infection, smoking, vitamin D insufficiency, and obesity during adolescence seem to have the strongest evidence of increasing the likelihood of developing MS (Olsson et al. 2017). Nonetheless, the major pathogenic mechanism is related to the migration of peripheral immune cells across the blood–brain barrier (BBB) into the CNS. Initially, this process was long attributed solely to autoreactive CD4⁺ T helper type 1 (Th1) cells (Fletcher et al. 2010). However, further research has highlighted the roles of other T cell subsets, such as Th17 and CD8⁺ T cells, as well as B cells, macrophages, and microglia (Cencioni et al. 2021). Altogether, these immune components initiate an autoimmune response which is believed to be directed against myelin sheaths antigens, such as myelin basic protein, leading to demyelination and, consequently, axonal degeneration (Martinsen and Kursula 2022; Mey et al. 2023). In addition to inflammatory mechanisms, emerging studies emphasize neurotransmitter dysregulation, glutamate-mediated excitotoxicity, as well as disturbances in acetylcholine and dopamine signaling, as a critical components of disease pathophysiology (Akyuz et al. 2023; Caligiore et al. 2025). Notably, acetylcholine and dopamine may modulate T cell-mediated inflammation. In mice, inhibition of cholinergic signaling via vagotomy has been shown to suppress CD4⁺ T cell proliferation, while in vitro, haloperidol, a dopamine D2 receptor antagonist, has been found to inhibit dendritic cell maturation and their capacity to induce T helper 1 (Th1) differentiation (Akyuz et al. 2023).

MS is diagnosed based on recently revised McDonald criteria, involving clinical and radiological features, but also laboratory biomarkers (Montalban et al. 2025). Clinically, it can be divided into three main disease courses: relapsing-remitting (RRMS), which is most common one, observed in over 80% of all, primary progressive (PPMS), characterized by gradual progression of neurological deficits, and secondary progressive (SPMS), defined as gradual worsening of disability after a relapsing course (Lublin et al. 2014). MS typically affects young adults between the ages of 20 and 40, with the incidence of the disease being approximately three times higher in females (Howard et al. 2016; Dobson and Giovannoni 2019). However, it is also observed in children, where is known as pediatric onset MS, and in adults aged over 50 years (Montalban et al. 2025). Due to multifocal damage to the CNS, which is referred to as dissemination in space, MS is a heterogenous disease, which might have various manifestations. The most common presenting symptoms include decreased visual acuity caused by optic neuritis, limb paresis, sensory disturbances, but also urinary incontinence (Oh et al. 2018). Additionally, MS might be associated with psychiatric symptoms, which may be caused either by the disease course itself or by the treatment agents used by clinicians, including glucocorticoids and interferon (IFN)-β (Cheema et al. 2019). Interestingly, psychiatric symptoms have been linked to MS since the first description of the disease by Jean-Martin Charcot in 1868 (Zalc 2018; Menculini et al. 2023). Currently, neuropsychiatric manifestations are reported in as high as 60% of all people with MS (PwMS), representing one of the major factors contributing to morbidity and mortality associated with the disease (Murphy et al. 2017). However, the vast majority of reports focus on depression and anxiety disorders (Jellinger 2024), while relatively little is known about psychosis in the course of MS (P-MS). Therefore, this narrative review aims to summarize the current knowledge regarding the prevalence, clinical manifestations, treatment approaches, and potential molecular pathomechanisms involved in P-MS.

## Methods: search strategy and selection criteria

To comprehensively examine the topic, the PubMed database was searched from inception to October 2025. Both experimental and clinical studies, including case reports and case series, were considered for inclusion. Titles and abstracts were screened searching for key terms such as ‘multiple sclerosis’, ‘psychopathology’, ‘psychosis’, ‘psychiatric symptoms’ or ‘psychiatric manifestations’, and ‘experimental autoimmune encephalomyelitis’, which were variously combined. Only articles published in English were selected. In addition to the primary search results, reference lists of relevant publications were manually reviewed to identify additional eligible studies. The search was conducted independently by two authors (P.O., A.P.).

## Prevalence and risk factors for psychosis in multiple sclerosis

Early analyses of psychosis in PwMS did not identify significant differences in its prevalence, suggesting rates comparable to those in the general population. However, given the fact that P-MS remains insufficiently studied, subsequent research indicates that its prevalence may be up to three times higher than in individuals without MS (Kosmidis et al. 2010). According to a study by Huang and colleagues, analyzing data from the Taiwan National Health Insurance Research Database, PwMS have a 2.574-fold increased risk of developing psychotic disorders (Huang et al. 2021).

The prevalence of P-MS has been shown to range between approximately 2% and 4% in the analyzed cohorts (Kosmidis et al. 2010; Murphy et al. 2017). Interestingly, a prospective study by Diaz-Olavarrieta et al., involving 44 clinically stable PwMS who did not receive glucocorticoids and 25 matched controls, revealed that psychiatric symptoms were observed in 95% of PwMS compared to only 16% in the control group. Additionally, up to 10% of PwMS experienced hallucinations and 7% of them reported delusions (Diaz-Olavarrieta et al. 1999). Furthermore, a meta-analysis by Ketata and co-workers, indicated that in 65.1% of cases, P-MS presented before or concomitantly with the diagnosis of MS. Moreover, the prevalence of psychotic disorders (51.1%) was higher than that of mood disorders (39.5%) (Ketata et al. 2025). Notably, in 59.4% of P-MS cases, psychosis was treatment-resistant, suggesting the importance of considering MS in the differential diagnosis of psychosis, particularly in individuals who are resistant to standard medications (Ketata et al. 2025).

Relatively little is known about the risk factors associated with psychiatric disorders in PwMS. Mado et al. suggested that social isolation and lack of family support can increase susceptibility to mental disorders. Furthermore, evidence suggests a link between disability level and neuropsychiatric conditions, as a significant correlation was found between the Expanded Disability Status Scale (EDSS) score and the occurrence of clinically diagnosed mental disorders over a lifetime (Mado et al. 2024). MS and schizophrenia are both conditions with ambiguous and complex etiologies, which still remain unclear (Misiak et al. 2023). Clinical and epidemiological features of both MS and psychotic disorders suggest potential common factors. Both conditions affect young adults, and their courses are characterized by periods of remissions and flare-ups (Arneth 2017; Inanc and Kaya 2022). Furthermore, studies have indicated a shared genetic background between the two conditions, for as many as 21 independent loci associated with schizophrenia are also linked to MS. In particular, certain alleles of human leukocyte antigens (HLAs) pathogenetically connect schizophrenia with MS (Khandaker et al. 2015; Arneth 2017). On the other hand, other alleles, including DRB103:01* and DQB102:01*, which are thought to increase the risk of MS, are conversely believed to decrease the risk of schizophrenia (Misiak et al. 2023). Additionally, the aforementioned genetic predispositions suggest that schizophrenia might be associated with immunological mechanisms. Nonetheless, the evidence regarding exact causal pathways are still lacking (Khandaker et al. 2015). The common overlapping neuroinflammatory pathways may potentially explain their clinical and biological convergence, including dysregulation of the immune response within the CNS, particularly through activation of microglia, as well as infiltration of peripheral immune cells (Khandaker et al. 2015; Misiak et al. 2023; Ermakov et al. 2025). Interestingly, treatment-naïve patients with schizophrenia experiencing their first psychotic episode, as well as those undergoing acute psychotic relapse, tend to have elevated serum levels of proinflammatory cytokines including interleukin (IL)-6, tumor necrosis factor (TNF)-α, IL-1β, and interferon (IFN)-γ, alongside reduced levels of the anti-inflammatory IL-10. Additionally, these immune alterations tend to normalize following symptom remission with antipsychotic therapy (Khandaker et al. 2015). Furthermore, up to 30% of patients with schizophrenia may show oligoclonal bands in their cerebrospinal fluid (Campana et al. 2022). On the contrary to PwMS, type 4 oligoclonal bands are the most common in this group (Campana et al. 2022), suggesting a systemic inflammatory process that also affects the CNS, rather than the intrathecal immunoglobulin synthesis typical of MS (Carta et al. 2022).

## Pathomechanism of MS-associated psychosis

While the precise mechanisms underlying psychotic syndromes in PwMS remain unclear, existing evidence suggests that these psychiatric manifestations may stem from structural or functional disruption of synaptic networks (Menculini et al. 2023). In combination with immune mechanisms, dysfunction of neurotransmission pathways may cause psychotic symptoms, especially given that at least some patients with psychosis may exhibit the presence of autoantibodies, typically directed against N-methyl-D-aspartate (NMDA) receptors (Arneth 2017). The increase of pro-inflammatory cytokines, involving TNF-α, within demyelinating lesions may reduce glutamate uptake by decreasing the activity of transporters such as GLT-1 and GLAST, while simultaneously increasing glutaminase activity in microglia, thereby leading to a focal increase in glutamate levels (Fairless et al. 2021). Therefore, the hypothesis of NMDA receptor hypofunction in schizophrenia intuitively suggests increased glutamate release in areas where glutamatergic neurons are disinhibited due to primary NMDA receptor hypofunction (Kruse and Bustillo 2022), potentially leading to excitotoxicity and neuronal degeneration. Paradoxically, this may manifest as cortical thinning observed in some patients with schizophrenia (Plitman et al. 2014). Neuroinflammation simultaneously redirects the kynurenine pathway towards increased quinolinic acid (QA) synthesis, accompanied by a corresponding decrease in kynurenine acid (KA) production (Lovelace et al. 2016; Kupjetz et al. 2025). Given that QA is a neurotoxin that promotes NMDA receptor-mediated glutamate excitotoxicity, while KA exerts antagonistic effects on both NMDA and α7 nicotinic acetylcholine receptors, this shift amplifies the excitotoxic consequences of neuroinflammation (Kupjetz et al. 2025). Furthermore, in vivo studies have shown that inhibition of a key enzyme in the kynurenine pathway, leading to a reduction in KA levels, causes a transient increase in dopamine release in the striatum (Wu et al. 2014). Collectively with the dopamine hypothesis of psychosis, which posits that excessive dopaminergic activity, particularly in the striatum, contributes to the manifestation of positive psychotic symptoms such as hallucinations and delusions (Howes and Kapur 2009), changes in the kynurenine pathway may represent a potential mechanistic link between MS and psychotic symptoms (Lovelace et al. 2016).

Interestingly, evidence highlights a correlation between specific locations of demyelinating lesions observed on magnetic resonance imaging (MRI) and the occurrence of P-MS. For instance, Feinstein et al., in a study involving 10 PwMS experiencing psychotic symptoms and 10 matched PwMS controls without psychiatric disorders, reported an increased total lesion load and higher periventricular lesion scores in the P-MS group (Feinstein et al. 1992). Also, there was a trend toward a higher total lesion load in the left trigone and regions surrounding the third ventricle in the P-MS cohort (Feinstein et al. 1992). It is in line with the case series by Gilberthorpe and colleagues, which has revealed that 11 out of 15 (73%) MRI scans showed lesions in the periventricular white matter. Additionally, four MRI reports (27%) indicated unilateral or bilateral involvement of the temporal lobes (Gilberthorpe et al. 2017). Moreover, a meta-analysis conducted by Ketata et al. demonstrated a significant association between the presence of demyelinating lesions in the periventricular white matter, cerebellar peduncles, and cerebellum on MRI and the development of psychosis in previously diagnosed PwMS (Ketata et al. 2025). Also, lesions located in the temporal lobes were more frequent findings in patients with visual hallucinations compared to those without (Ketata et al. 2025). Additionally, a systematic review of PwMS with psychotic symptoms reported that 60% had predominantly fronto-temporal lesions, and 75% exhibited contrast-enhancing lesions, indicating inflammatory activity (Camara-Lemarroy et al. 2017).

Interestingly, studies have shown that individuals with schizophrenia might exhibit structural brain abnormalities, including fronto-temporal atrophy (Olabi et al. 2012). Finally, fMRI studies in schizophrenia have demonstrated reduced fronto-temporal connectivity associated with auditory hallucinations (Lawrie et al. 2002). The presence of demyelinating lesions in these regions of PwMS may similarly disrupt neural communication within these networks, potentially contributing to psychotic symptoms. However, establishing a definitive causal link warrants further investigation.

## The clinical manifestations of psychosis in multiple sclerosis

The term ‘psychosis’ does not yet have a single, accepted definition, but refers to a clinical syndrome that encompasses a diverse range of symptoms (Gaebel and Zielasek 2015). It is characteristic of many medical, neurological, neurodevelopmental, and psychiatric disorders and is therefore a key focus of assessment and care in neurological and psychiatric practice (Arciniegas 2015). Both the American Diagnostic and Statistical Manual of Mental Disorders (DSM)-5 and the International Classification of Diseases 11th Revision (ICD-11) do not specifically describe ‘psychosis’; instead, they describe ‘schizophrenia spectrum and other psychotic disorders’ (DSM-5) or ‘schizophrenia and other primary psychotic disorders’ (ICD-11). In the DSM-5, psychotic disorders are defined by abnormalities in one or more of the following five domains: delusions, hallucinations, disorganized thinking (speech), grossly disorganized or abnormal motor behavior (including catatonia), and negative symptoms, while in the ICD-11, these disorders are characterized by impairments in reality testing and alterations in behavior that manifest in positive symptoms (persistent delusions, persistent hallucinations, disorganized thinking, grossly disorganized behavior, and experiences of passivity and control), negative symptoms such as blunted or flat affect and avolition, and psychomotor disturbances. For greater clarity, the DSM-5 defines negative symptoms as being more prominent in schizophrenia than in other psychotic disorders, particularly two key symptoms: diminished emotional expression and avolition. The former refers to ‘reductions in the expression of emotions’, while the latter is ‘a decrease in motivated, self-initiated, purposeful activities. Given the variety of symptoms that make up psychosis, it has been proposed to break the term down into its individual symptoms (Gaebel and Zielasek 2008). A five-factor solution that includes hallucinations, delusions, disorganization, excitement, and emotional distress is typically the result of factor analyses of psychotic symptoms in severe mental disorders, such as schizophrenia (VANDERGAAG et al. 2006). Depressive and manic symptoms also play a role when considering psychotic symptoms in the general population. This is because affective and other mental disorders often exhibit the key symptomatic features of psychosis (MURRAY et al. 2005).

### Disease-related psychotic symptoms in multiple sclerosis

P-MS exhibits some clinical features that can help to set it apart from typical psychotic disorders and that emphasize the intricate relationship between neurological and psychiatric symptoms (Gilberthorpe et al. 2017; Kim et al. 2024). While psychosis is frequently linked to primary psychiatric disorders like schizophrenia (Arciniegas 2015), the symptoms of P-MS are strictly connected with the disease’s neurodegenerative processes (Gilberthorpe et al. 2017). Several MRI studies have identified these symptoms as being associated with lesions in the frontotemporal region, medial temporal lobe, and periventricular white matter. Research has also indicated that the variety of psychotic symptoms experienced by PwMS, such as delusions, hallucinations, and mood disorders, are thought to be connected with demyelination and inflammatory processes that impact brain areas involved in mood regulation and perception (Camara-Lemarroy et al. 2017; Gilberthorpe et al. 2017).

P-MS can manifest clinically in a wide range, including schizophrenia-like syndromes and mood disorders with psychotic features (Table 1). Positive symptoms, such as persecutory delusions and hallucinations, tend to predominate in P-MS, in contrast to typical psychotic disorders, where negative symptoms like anhedonia and social withdrawal are more common (Camara-Lemarroy et al. 2017; Gilberthorpe et al. 2017). Among PwMS, delusions of paranoia or somatic delusions are frequently accompanied by neurological symptoms like cognitive impairment or abnormalities in gait (Özcan et al. 2014; Enderami et al. 2018; Kim et al. 2024). The clinical picture may become more complex if psychotic symptoms appear alongside or even before neurological symptoms. According to a systematic review by Sabe & Sentissi, approximately 20% of patients presented with psychosis before receiving a diagnosis of multiple sclerosis, which suggests that psychosis may be a prodromal symptom to the illness (Sabe and Sentissi 2022; Spiegel et al. 2023). Compared to primary psychotic disorders, P-MS usually has a better prognosis, with a faster resolution and fewer relapses (Haussleiter et al. 2009). This symptomatology emphasizes how important it is for medical professionals to take the neurological background into account when assessing mental health symptoms in PwMS (Kim et al. 2024).

**Table 1:** Clinical manifestations of psychosis in multiple sclerosis

Domain	Clinical features	Key characteristics in *P*-MS	Diagnostic relevance
Positive symptoms	Delusions	Predominant feature in P-MS	Frequently coexist with neurological deficits
	Hallucinations	Common but variable	May reflect lesions in temporal regions
	Disorganized thinking/speech	Present but less prominent than in primary psychosis	Can overlap with cognitive impairment
Negative symptoms	Blunted/flat affect	Less prominent than in Schizophrenia	Helps differentiate P-MS from primary psychotic disorders
	Avolition	Mild or secondary to cognitive decline	Not a dominant feature
Cognitive impairment	Memory deficits, reduced processing speed, executive dysfunction	Very common (up to 70% of patients)	May mimic or exacerbate psychiatric symptoms
Mood symptoms	Depression, mania with psychotic features	Frequently co-occur	Suggests overlap with affective disorders
Behavioral and psychomotor	Disorganized behavior, agitation, psychomotor disturbances	Variable presentation	Can be confused with neurological disability
Diagnostic challenges	Overlap with depression, anxiety, cognitive decline	High misdiagnosis risk	Requires integration of neurological and psychiatric assessment

*P-MS* psychosis in the course of multiple sclerosis

Differentiating P-MS from other neuropsychiatric disorders poses significant diagnostic challenges. Misdiagnosis may result from the overlap of psychiatric symptoms, including depression, anxiety, and cognitive dysfunction, with those of primary psychiatric disorders (Gilberthorpe et al. 2017). According to a study that used the neuropsychiatric inventory, even in cases where the disease was mild or in its early stages, up to 95% of PwMS experienced some kind of neuropsychiatric symptoms (Diaz-Olavarrieta et al. 1999). The diagnostic procedure is also made more difficult by the fact that P-MS can manifest either as primary psychotic disorders or as mood disorders with psychotic features (Vesic et al. 2023). Furthermore, psychosis can appear as an early MS symptom, sometimes months or even years before neurological symptoms, making the diagnosis ever more sophisticated (Sabe and Sentissi 2022). Between 30% and 70% of patients with MS experience cognitive impairment at some point during the course of their illness, which can further complicate the clinical picture (Rao et al. 1991). It can be challenging for clinicians to determine whether these behaviors are the result of underlying neurological damage or an independent psychiatric disorder, also because cognitive decline can mimic or worsen psychiatric symptoms (Kim et al. 2024).

The diagnostic standards for P-MS are still not entirely clear. To find lesions linked to psychotic symptoms, clinicians frequently use neuroimaging methods like MRI. Physicians frequently use the patient’s history and symptoms to determine whether the onset of psychosis corresponds with the neurological alterations typical for multiple sclerosis (Camara-Lemarroy et al. 2017). Steroid responsiveness has also been identified as a possible indicator for differentiating between psychotic types in this population. After receiving corticosteroid treatment during acute exacerbations, many patients report a notable improvement in their psychotic symptoms (despite psychosis being a known adverse effect of corticosteroids) (Camara-Lemarroy et al. 2017; Sabe and Sentissi 2022; Spiegel et al. 2023). According to a comprehensive epidemiological study conducted in Canada with over 10,000 MS patients, 2–4% of them had psychotic symptoms and satisfied the requirements for a psychotic disorder diagnosis (Patten et al. 2005). These symptoms frequently appear in people who have never experienced psychiatric illness before, which raises the possibility that they are caused by the neurological foundations of MS rather than underlying mental health issues (Gilberthorpe et al. 2017).

### Therapy-related psychotic symptoms in multiple sclerosis

Moreover, MS treatments like IFN-ß and corticosteroids can contribute to the development of psychosis (Kim et al. 2024). The fact that certain MS treatment methods, like glucocorticoids, can cause or aggravate psychiatric symptoms, resulting in a feedback loop that makes treatment plans more difficult, adds to this diagnostic ambiguity (Camara-Lemarroy et al. 2017; Gilberthorpe et al. 2017). Few studies have examined how corticosteroid therapy affects psychosis in PwMS to date, and it is still unknown which risk factors are most predictive of psychiatric changes (Vesic et al. 2023). These side effects are linked to long-term corticosteroid use, as well as increases in dosage and treatment duration (Vesic et al. 2023). According to multiple reports, using high doses of corticosteroids may result in psychiatric side effects such as depression, psychosis, suicidal thoughts, and hypomania/mania (Brown and Chandler 2001; Chwastiak and Ehde 2007). High-dose corticosteroid treatment may also intensify mood swings (Morrow et al. 2015). Pulse steroid treatment has been linked to mood disorders and depression in PwMS, raising the possibility that the patient’s psychiatric history may be a contributing factor (Lorefice et al. 2018; Vesic et al. 2023).

Immunomodulatory drugs may help bring MS into remission, but they may also directly contribute to the emergence of psychotic symptoms in certain situations (Vesic et al. 2023). Eleven PwMS without a history of mental illness who were taking IFN-β showed signs of severe depression and suicidality (Fragoso et al. 2010). A complex mood-behavior disorder linked to IFN-β use was evident in these patients’ phobic, aggressive, behavioral, psychotic, and manic symptoms. After stopping IFN-β, there was a complete remission of mental health issues (Fragoso et al. 2010). If psychosis emerges during INF therapy, discontinuing or reducing the INF dose is critical, as symptoms often resolve after cessation. Quetiapine may also be helpful in IFN-induced prolonged psychosis (Cheng et al. 2009; Fragoso et al. 2010). Due to the higher risk of extrapyramidal reactions in these patients, psychosis management typically involves antipsychotics at the lowest dose possible with negligible antidopaminergic effects (Marinho et al. 2017). A case of IFN-induced psychosis that was effectively managed with paliperidone has also been reported (Carrillo de Albornoz Calahorro et al. 2019).

To find out if using disease-modifying treatments is linked to negative psychiatric effects in MS patients, Gasim et al. carried out a systematic review. Natalizumab, fingolimod, dimethyl fumarate, teriflunomide, and alemtuzumab were all included in this study, which demonstrated that using them did not raise the risk of negative psychiatric effects in MS patients. Fingolimod even decreased the incidence of depressive symptoms (Gasim et al. 2018; Vesic et al. 2023). Furthermore, Krivinko et al. recently showed that treating rodents with fingolimod reduced behavioral abnormalities linked to psychosis (Krivinko et al. 2022; Vesic et al. 2023).

## Management strategies

The treatment of P-MS poses a particular set of difficulties, especially when it comes to the available pharmacological options (Davids et al. 2004). Since psychotic symptoms can be a direct result of MS or a side effect of medications used to treat it (Vesic et al. 2023), it is essential to comprehend the safety and effectiveness issues surrounding different pharmacological treatments in order to maximize patient outcomes (Davids et al. 2004). Evidence from a retrospective case series involving 13 patients diagnosed with P-MS suggested that atypical antipsychotics were the most frequently chosen option among clinicians. Specifically, 27% of patients received risperidone, 20% received olanzapine, while quetiapine and aripiprazole were each prescribed in 13% of cases (Gilberthorpe et al. 2017). While this recent data supports the use of atypical antipsychotics in P-MS, it should be noted that the available literature evidence is limited (Hussain and Belderbos 2008; Mitra et al. 2024). In addition, several references may not fully reflect current clinical practices or recent pharmacological developments, highlighting the need for updated research. Table 2 summarizes the psychopharmacological agents currently used in P-MS treatment.

When treating P-MS, a variety of antipsychotic agents are used, with differing degrees of success (Gilberthorpe et al. 2017). Atypical antipsychotics, like risperidone, olanzapine, quetiapine, and aripiprazole are frequently chosen because of their better side effect profiles when compared to conventional antipsychotics (Safferman et al. 1994; Davids et al. 2004; Hussain and Belderbos 2008; Lo Fermo et al. 2010; Zhang et al. 2013; Mitra et al. 2024).

However, individual patient responses and possible side effects must be taken into account when selecting an antipsychotic. For example, because clozapine has a lower incidence of extrapyramidal side effects (EPS), which can be especially problematic for MS patients who may already be experiencing motor symptoms associated with their condition, it has been promoted as a treatment option despite its adverse effects (Davids et al. 2004; Toyooka et al. 2013).

Regarding alternative options, in the case series by Gilberthorpe et al., 13% of patients with P-MS who were refractory to initial therapy were treated with clozapine. In one case, clozapine alone was insufficient to control symptoms, necessitating augmentation with amisulpride and lamotrigine (Gilberthorpe et al. 2017). Additionally, a separate case report described a patient in whom other antipsychotics were either ineffective or poorly tolerated; in this instance, ziprasidone was reported to be both effective and well tolerated (Davids et al. 2004).

The results of the numerous studies, that are part of a systematic review by Stamoula et al., support the idea that atypical antipsychotics not only help with psychotic symptoms but also have positive effects on neuroinflammation, which is important for MS treatment (Stamoula et al. 2022). Several other studies and case reports have confirmed the effectiveness of antipsychotic drugs in treating psychosis associated with multiple sclerosis (Kosmidis et al. 2010; Vesic et al. 2023). In certain patients, corticosteroids, which are frequently used to treat acute relapses of multiple sclerosis (Pozzilli et al. 2004), have been shown to reduce psychotic symptoms (Camara-Lemarroy et al. 2017). A patient with MS-related psychosis showed notable improvement following corticosteroid treatment, according to a case study, indicating that the anti-inflammatory qualities of these medications may also help with psychiatric symptoms (Spiegel et al. 2023). But because corticosteroids have the potential to cause or worsen psychosis in vulnerable people, their use is debatable (Kim et al. 2024). The Maudsley guideline suggests that the advantages of corticosteroid therapy might exceed the risks, notwithstanding this danger (Taylor et al. 2021). According to a systematic review including 32 cases, immunosuppressive treatment was substantially more successful than antipsychotics in treating psychotic symptoms after an MS diagnosis (Sabe and Sentissi 2022). Despite these encouraging results, little empirical evidence has been found about the optimal pharmacological management practices for P-MS (Painold et al. 2011).

When prescribing pharmacological treatments for P-MS, safety considerations are crucial. The underlying neurological condition may increase the risk of adverse drug reactions. Atypical antipsychotics, for instance, have been linked to metabolic syndrome conditions, which can make the clinical picture more complicated for PwMS who are already at risk because of their immobility or other disease-related factors (Carli et al. 2021; Gauthier et al. 2024). Furthermore, there is evidence that suggests PwMS may be more sensitive to some antipsychotic drugs like clozapine and risperidone. Therefore, it is advised to titrate atypical anti-psychotics gradually over a period of two weeks until the therapeutic dose is achieved (La Flamme et al. 2020).

When managing psychiatric symptoms induced by corticosteroids, the general treatment approach involves initially reducing the dosage or discontinuing the corticosteroid altogether (Vesic et al. 2023). Antipsychotics are commonly employed, with haloperidol being the most frequently prescribed (Huynh and Reinert 2021). Because of its known adverse event profile, which was documented in the Silva and Tolstunov case, haloperidol should not be prescribed universally or arbitrarily in the treatment of steroid-induced psychosis, even though it has a proven track record of success (Silva and Tolstunov 1995; Huynh and Reinert 2021). Second-generation drug risperidone has also been shown to be effective in a limited number of patient cases and studies (Huynh and Reinert 2021). Additionally, quetiapine has demonstrated efficacy in lowering psychological distress and irritability in patients suffering from steroid-induced psychosis (Hrebicek K and Olinka MIN 2021). Since atypical antipsychotics have a better tolerability profile, are less likely to impact the extrapyramidal system, and lower the risk of developing pseudoparkinsonism and catalepsy, they continue to be the recommended course of treatment for these patients (Taylor et al. 2021). Although first-generation antipsychotics such as haloperidol are still widely used, evidence also supports the potential effectiveness of lithium, selective serotonin reuptake inhibitors (SSRIs), tricyclic antidepressants (TCAs), second-generation antipsychotics, and certain antiepileptic drugs, including carbamazepine and valproic acid and its derivatives. However, current data remain insufficient to establish a standardized treatment protocol for affected patients (Goggans et al. 1983; Brown et al. 1999; Bhangle et al. 2013; Huynh and Reinert 2021).

It is crucial to consider both pharmaceutical and non-pharmacological options when talking about P-MS. Several studies advocate combining these strategies to enhance patient outcomes. Cognitive-behavioral therapy (CBT), family therapy, and other psychosocial interventions have demonstrated benefits in the management of schizophrenia symptoms. These therapies help patients cope with symptoms, improve insight, and enhance social functioning (Nasim et al. 2025). Cognitive remediation programs, which are structured training-based behavioral interventions, can improve cognitive impairments, leading to better psychosocial functioning (Nibbio Gabriele et al. 2024). Through the modulation of cortical excitability, techniques like transcranial magnetic stimulation and transcranial direct current stimulation have demonstrated promise in improving mental and cognitive symptoms, including those associated with psychosis (Nibbio Gabriele et al. 2024). Some case reports describe the use of plasma exchange in patients experiencing psychotic reactions as a manifestation of MS relapse (Gabelić et al. 2012; Nandy et al. 2020).

**Table 2:** Psychopharmacological treatments used in multiple sclerosis-related psychosis

Medication group	Examples	Indications	Benefits	Side Effects	Evidence
Atypical antipsychotics	Risperidone, Olanzapine, Quetiapine, Aripiprazole, Ziprasidone	First-line treatment of P-MS; clozapine and ziprasidone used as second-line treatment; risperidone used in steroid-induced psychosis; quetiapine and paliperidone used in IFN-induced psychosis	Effective; lower EPS risk; some may reduce neuroinflammation; quetiapine lowers irritability	Metabolic syndrome; some require slow titration; clozapine may need augmentation	Safferman et al. (1994); Silva and Tolstunov (1995); Davids et al. (2004); Hussain and Belderbos (2008); Cheng et al. (2009); Kosmidis et al. (2010); Lo Fermo et al. (2010); Fragoso et al. (2010); Toyooka et al. (2013); Zhang et al. (2013); Gilberthorpe et al. (2017); Carrillo de Albornoz Calahorro et al. (2019); La Flamme et al. (2020); Hrebicek K and Olinka MIN (2021); Carli et al. (2021); Huynh and Reinert (2021); Stamoula et al. (2022); Vesic et al. (2023); Mitra et al. (2024); Gauthier et al. (2024)
First-generation antipsychotics	Haloperidol	Steroid-induced psychosis; older treatment options	Proven efficacy	High risk of EPS, catalepsy, and pseudoparkinsonism; should not be used arbitrarily	Silva and Tolstunov (1995); Huynh and Reinert (2021)
Lithium	Lithium	Prophylactic and acute treatment of steroid-induced psychosis; management of steroid-induced severe mood disorders;	May have a neuroprotective effect	Requires monitoring for toxicit; renal dysfunction or alterations in leukocyte counts may limit the use	Brown et al. (1999); Bhangle et al. (2013); Huynh and Reinert (2021)
Antiepileptics	Carbamazepine, Valproic Acid	Treatment and prevention of steroid-induced psychiatric symptoms; augmentation in resistant psychosis	Mood-stabilizing effects; cognitive protection;	Interaction with corticosteroids	Brown et al. (1999); Bhangle et al. (2013); Huynh and Reinert (2021)
SSRIs / TCAs	Fluoxetine, Amitriptyline	Withdrawal and depressive symptoms during corticosteroids therapy	Mood-stabilizing effects	May exacerbate mood symptoms; the use of tricyclic antidepressants in treating steroid-induced psychosis is described as controversial	Brown et al. (1999); Bhangle et al. (2013); Huynh and Reinert (2021)

## Conclusion

PwMS have a higher risk of developing psychosis than the general population. Psychosis can occur before, at the time of, or later in the course of MS diagnosis. While the exact pathomechanism is still uncertain, it may involve inflammatory processes, neurotransmitter alterations, and structural brain changes due to demyelinating lesions in specific regions. Moreover, some MS treatments, including glucocorticoids and IFN-β, may trigger psychotic symptoms. Therefore, neurologists should be prepared to manage P-MS, and on the other hand MS should be considered in patients with a first psychotic episode that does not respond to standard antipsychotic treatments.

## Data Availability

No datasets were generated or analysed during the current study.
